# Engineering of chitosan and collagen macromolecules using sebacic acid for clinical applications

**DOI:** 10.1186/2194-0517-2-11

**Published:** 2013-04-23

**Authors:** G Sailakshmi, Tapas Mitra, A Gnanamani

**Affiliations:** Microbiology Division, Central Leather Research Institute (CSIR, New Delhi), Adyar, Chennai, 20 Tamil Nadu India

**Keywords:** Chitosan, Collagen, Sebacic acid, Mechanical strength, Biocompatible

## Abstract

Transformation of natural polymers to three-dimensional (3D) scaffolds for biomedical applications faces a number of challenges, *viz*., solubility, stability (mechanical and thermal), strength, biocompatibility, and biodegradability. Hence, intensive research on suitable agents to provide the requisite properties has been initiated at the global level. In the present study, an attempt was made to engineer chitosan and collagen macromolecules using sebacic acid, and further evaluation of the mechanical stability and biocompatible property of the engineered scaffold material was done. A 3D scaffold material was prepared using chitosan at 1.0% (*w*/*v*) and sebacic acid at 0.2% (*w*/*v*); similarly, collagen at 0.5% (*w*/*v*) and sebacic acid at 0.2% (*w*/*v*) were prepared individually by freeze-drying technique. Analysis revealed that the engineered scaffolds displayed an appreciable mechanical strength and, in addition, were found to be biocompatible to NIH 3T3 fibroblast cells. Studies on the chemistry behind the interaction and the characteristics of the cross-linked scaffold materials suggested that non-covalent interactions play a major role in deciding the property of the said polymer materials. The prepared scaffold was suitable for tissue engineering application as a wound dressing material.

**Electronic supplementary material:**

The online version of this article (doi:10.1186/2194-0517-2-11) contains supplementary material, which is available to authorized users.

## Background

Polymers, in general, (whether natural or synthetic) play major roles in biomaterial preparations. However, natural polymers or polymers derived from living creatures are of greater interest, and most research publications majorly discussed chitosan and collagen Parenteau-Bareil et al. (2010; Iwasaki et al. 2011). Further, Ko et al. (2010) suggested the use of naturally derived polymers as three-dimensional (3D) scaffolds, which received much attention due to their low cost, ease of processing, and biocompatibility.

With regard to chitosan and collagen, after extraction, both these materials did not have much stability to act as a biomaterial for clinical applications and demanded stabilizers in the form of cross-linkers Friess (1998; Austero et al. 2012). Diisocyanates, Resimene Ligler et al. (2001), *N*, *N*-disuccinimidyl suberate Schauer et al. (2003), epichlorohydrin Wei et al. (1992), Genipin Jin et al. (2004), and glutaraldehyde Tual et al. (2000) were the stabilizers studied for chitosan and chromium Usha and Ramasami (2000), while aldehydes Sheu et al. (2001), hexamethylene diisocyanate Miles et al. (2005), carbodiimide Nam et al. (2008), acyl azides Petite et al. (1990), citric acid, maleic acid derivatives Saito et al. (2008), and various other physical treatments, such as UV Weadock et al. (1995) and gamma irradiation Olde Damink et al. (1995a), were the stabilizers studied for type I collagen. All the said exogenous cross-linkers cross-linked with chitosan or with type I collagen through (1) covalent amide/imine linkage, (2) metal-protein complex formation (chromium cross-linking with type I collagen), and (3) H-bond formation (between polyphenolic -OH group with different types of amino acids of type I collagen molecule and amino group of chitosan). Although the resultant biomaterial upon cross-linking with these agents was acceptable, the complete utilization of these materials was restricted because of low mechanical strength Schiffman and Schauer (2007). The biocompatibility of the scaffold material is also questionable because of the release of toxic components from some of the cross-linkers upon usage Gough et al. (2002).

In general, the mechanical property of any biomaterial depends on the interaction between the cross-linkers/stabilizers and the parent molecule (here, it is chitosan and collagen type I) Rinaudo (2010). As summarized above, reports on bonding interaction of the said cross-linkers suggest the predominance of covalent interactions which ultimately restrict the molecule to attain the desired mechanical strength.

Hence, in order to engineer the macromolecules and also to obviate the problems associated with the mechanical property and biocompatibility of biomaterials, we attempted to cross-link the parent molecule with a suitable cross-linker through non-covalent interactions. In our previous study, we detailed the cross-linking chemistry between malonic acid (MA) with chitosan/collagen Mitra et al. (2012). Observations with short-chain dicarboxylic acid (MA, three carbons) cross-linked chitosan/collagen scaffold gave impetus to carry out further work with long-chain dicarboxylic acids, namely sebacic acid (SA) (ten carbons).

Thus, in the present study, SA was chosen to cross-link with natural polymers, and to this day, no reports are available on the non-covalent interaction of SA with chitosan and type I collagen. Sebacic acid (decanedioic acid), a C-8 dicarboxylic acid (HOOC-(CH_2_)_8_-COOH), is a white flake or powdered crystal generally used as a component in metalworking fluids, surfactants, lubricants, detergents, oiling agents, emulsifiers, etc. Sebacic acid is the natural metabolic intermediate in *ω*-oxidation of medium- to long-chain fatty acids Liu et al. (1996). According to Tamada and Langer (1992), it is safe under *in vivo* condition.

The present study emphasizes the detailed chemistry behind the engineering of chitosan and collagen using SA and the evaluation of the thermal, mechanical, and biocompatible properties of the engineered chitosan and collagen scaffolds.

## Results and discussion

### Scaffold preparation using sebacic acid: understanding the cross-linking chemistry

For the preparation of any scaffold materials, the solution form of the parent compound/polymer is required to proceed further. However, in the case of chitosan and collagen, acetic and formic acids were generally used for dissolution Ohkawa et al. (2004; El-Tahlawy et al. 2006). The ‘proton exchange’ between the -COOH groups of acid molecule and free -NH_2_ groups of chitosan and collagen could be the reason for the dissolution in the said acids.

**Scheme 1:**
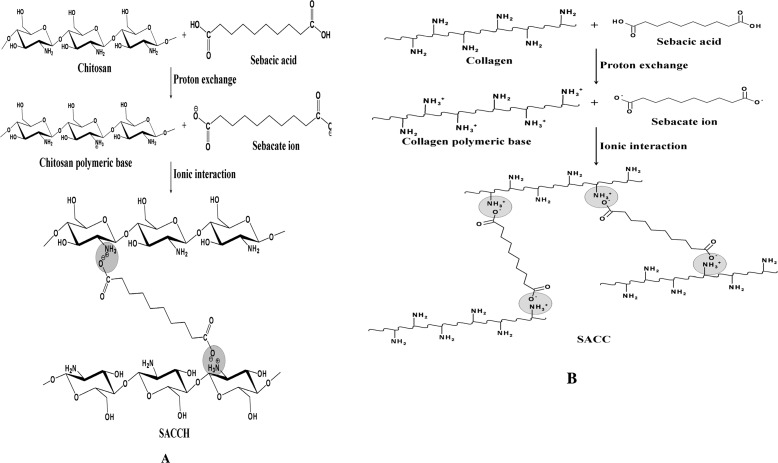
**Possible reaction mechanisms.** (**A**) Possible reaction mechanism between chitosan and sebacic acid. (**B**) Possible reaction mechanism between collagen and sebacic acid.

Therefore, it has been expected that like acetic acid, sebacic acid is also able to donate protons to dissolve chitosan and type I collagen. Further, similar to the interaction of TPP Bhumkar and Pokharkar (2006) with chitosan, sebacic acid may also interact with both natural polymers through ionic interaction. Because of the said proton exchange, chitosan and type I collagen dissolve in the presence of sebacic acid in water; the following schematic representation (Scheme 1a,b) illustrates the nature of proton exchange between sebacic acid with chitosan and with collagen for better understanding.

**Figure 1:**
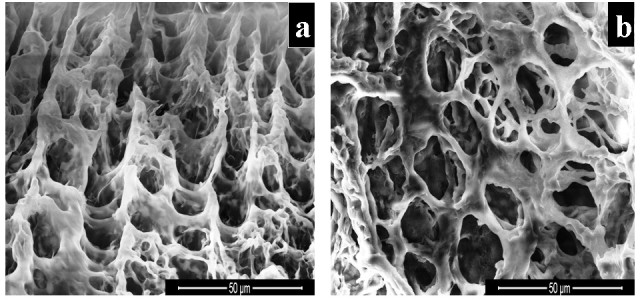
**SEM micrographs of (a) sebacic acid cross-linked chitosan and (b) sebacic acid cross-linked collagen scaffolds.**

Because of the said interaction, both natural polymers were completely dissolved in water in the presence of sebacic acid. With the resulting solution, scaffolds were prepared and subjected to characterization studies. Figure 1 shows the morphological features of the cross-linked scaffolds, namely sebacic acid cross-linked chitosan (SACCH) and sebacic acid cross-linked collagen (SACC). The 3D scaffold material was highly porous, and the pore structures of the membranes were well distributed and interconnected. It was obvious that most of the membrane volume was taken up by interconnecting pore space. The high porosity suggests the suitability of this scaffold for biomedical applications, including serving as absorption sponges and matrices for cell proliferation.

**Figure 2:**
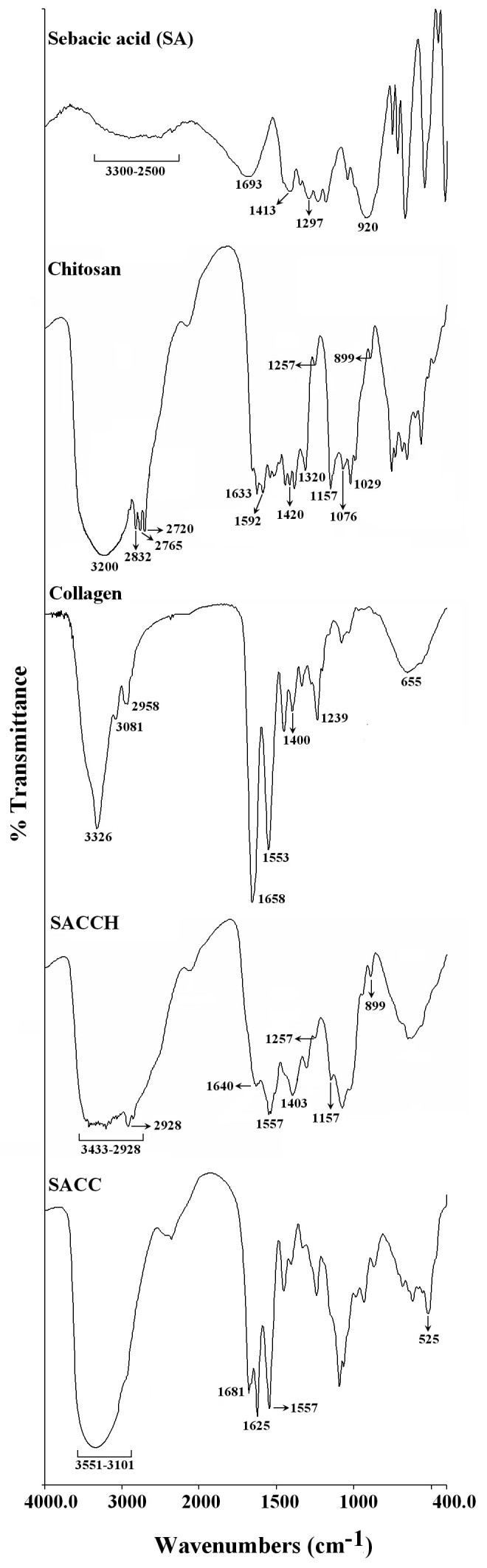
**FT-IR spectra of SA, chitosan, type I collagen, SACCH, and SACC scaffolds.** SA, sebacic acid; SACCH, sebacic acid cross-linked chitosan; SACC, sebacic acid cross-linked collagen.

Fourier transform infrared spectroscopy (FT-IR) studies were conducted to monitor chemical modifications in the chitosan and collagen structures upon cross-linking with SA. Figure 2 illustrates the FT-IR spectral details of SA, chitosan, collagen, SACCH, and SACC. Table 1 demonstrates the FT-IR peak assignments of SA, chitosan, and collagen. In the SACCH spectrum, few significant changes were observed. A broad, strong absorption peak in the region of 3,433 to 2,928 cm^-1^ resulted from the superimposed -OH and -NH_3_^+^ stretching bands. Absorption in 1,640 and 1,557 cm^-1^ corresponded to the presence of asymmetric N-H (-NH_3_^+^) bends and asymmetric -COO^-^ stretching, respectively. A peak observed at 1,403 cm^-1^ was due to symmetric -COO^-^ stretching. Other absorption peaks around 1,257, 1,157, and 899 cm^-1^ observed in the SACCH spectrum were similar to the native chitosan spectrum which exhibits that there was no change in the main backbone of the chitosan structure Lopez et al. (2008).

**Table 1:** **FT-IR analysis of SA, chitosan, and collagen**

Material	Wave number (cm^-1^)	Peak assignment
SA	3,300 to 2,500	Overlapping stretching vibration of C-H and O-H groups (*ν*_C-H_-*ν*_OH_)
	1,693	-C=O group (*ν*_C=O_)
	1,413	C-O-H in-plane bending ( *δ*_C-O-H_)
	1,297	C–O stretching vibration (ν_C-O_)
	920	Out-of-plane bending of the bonded O-H (*δ*_O-H_)
Chitosan	3,200	-NH_2_ stretching vibration (*ν*_NH_)
	2,832, 2,765, 2,720	Symmetric or asymmetric -CH_2_ stretching vibration attributed to the pyranose ring (*ν*_C-H_)
	1,633	-C=O in acetamide group (amide I band)
	1,592	-NH_2_ bending vibration in amino group (*δ*_NH_)
	1,420, 1,320	Vibrations of OH, CH in the ring
	1257	C-O group
	1157	-C-O-C in the glycosidic linkage
	1,076, 1,029	C-O stretching in acetamide (*ν*_C-O_)
	899	Corresponds to the saccharide structure
Collagen	3,326	-NH_2_ stretching vibration (*ν*_NH_)
	3,081	Fermi resonance overtone of 1,553 band
	2,958	C-H stretching (*ν*_C-H_)
	1,658	Amide I band (*ν*_C=O_)
	1,553	Amide II band (*δ*_NH_)
	1,400	Amide III band (*ν*_C-N_)
	1,239	C-N stretching of amine (*ν*_C-N_)
	655	Out-of-plane N-H wagging of amide and amine (*δ*_NH_)

*ν*, stretching; *δ*, bending.

In the SACC spectrum, few changes were observed when compared with native type I collagen. A broad, strong absorption peak in the region of 3,551 to 3,101 cm^-1^ resulted from the superimposed -OH and -NH_3_^+^ stretching bands. In the type I collagen spectrum, a sharp intense amide I band observed around 1,658 cm^-1^ disappeared with the appearance of two new bands in 1,681 and 1,625 cm^-1^ in the SACC spectrum; these bands were supposed to be caused by -NH_3_^+^ and -COO^-^, respectively. Moreover, when compared with native type I collagen spectrum, there was a reduction in the region of 1,557 cm^-1^ (overlapped band of amide II and free primary amines) in the SACC spectrum, which may be due to the reduction of free -NH_2_ group in the SACC. In the SACC spectrum, the observed band around 525 cm^-1^ was ascribed to the N-H oscillation of -NH_3_^+^. Results from FT-IR analysis reflected that SA was ionically cross-linked with chitosan and type I collagen Pavia et al. (2001; Lawrie et al. 2007).

Though FT-IR analysis displayed the ionic interaction between the cross-linker and the natural polymers, results on the percentage of cross-linking degree calculations suggested that increasing the concentration of SA increases the degree of cross-linking up to 0.4% and confirmed the interaction. About 60% to 65% cross-linking was observed with 0.2% SA with chitosan and collagen. However, in the case of experiments with glutaraldehyde, about 88% to 93% of cross-linking was observed with 0.2% concentration.

With regard to the mechanical property of the scaffold materials, it is a fundamental property for any scaffold material in the application point of view. From the results, we observed that the mechanical strength of the scaffold increased with the increase in sebacic acid concentration up to 0.2%. Further increase in SA concentration leads to the decrease in mechanical strength (results not shown). Table 2 illustrates the tensile strength, Young's modulus, and stiffness of native and sebacic acid (0.2%) cross-linked scaffolds. High tensile strength (MPa) values were observed for both the cross-linked scaffolds (SACCH 8.94; SACC 2.96) than for the native polymers (chitosan 0.37; type I collagen 0.13). Moreover, the Young's modulus of SACCH and SACC were 200.8 and 18.61, respectively. The stiffness values (SACCH 9.53 N/mm; SACC 0.69 N/mm) were also greater than those of the native polymers (chitosan 0.79 N/mm; type I collagen 0.3 N/mm).

**Table 2:** **Assessment of mechanical properties of chitosan, SACCH, collagen and SACC**

Samples	Maximum load^a^	Tensile strength^a^	Elongation at break^a^	Extension at maximum load^a^	Young's modulus/tensile modulus^a^	Stiffness ( ***κ***)^a^
	(N)	(MPa)	(%)	(mm)	(MPa)	(N/mm)
Chitosan	1.32 ± 0.08	0.37 ± 0.03	8.33 ± 0.6	1.67 ± 0.04	4.43 ± 0.5	0.790 ± 0.07
SACCH	5.61 ± 0.5	3.21 ± 0.2	8.63 ± 1	1.73 ± 0.04	37.1 ± 2.2	3.24 ± 0.4
Collagen	0.37 ± 0.03	0.13 ± 0.02	6.17 ± 0.8	1.23 ± 0.05	2.1 ± 0.7	0.3 ± 0.01
SACC	2.22 ± 0.5	2.96 ± 0.5	31.84 ± 2	3.18 ± 0.4	18.61 ± 1.5	0.698 ± 0.02

The assessment is in terms of tensile strength, elongation at break, Young's modulus, and stiffness. ^a^Mean ± SD

All these observations on mechanical properties suggest that sebacic acid cross-linked materials demonstrated appreciable mechanical strength compared to glutaraldehyde, wherein we observed brittleness similar to the observation made by Schiffman and Schauer (2007). Further, when the concentration of SA was increased > 0.2%, a decrease in mechanical strength was observed, and this could be reasoned to the high degree of cross-linking of SA with the polymers, as evidenced from the 2,4,6-trinitrobenzenesulfonic acid (TNBS) assay Wang et al. (2002).

Further, the reason for the brittle nature of the glutaraldehyde cross-linked material is that glutaraldehyde could covalently cross-link with chitosan and collagen through the formation of a double bond (C=N, imine bond) between the -CHO group of glutaraldehyde and the -NH_2_ group of natural polymers (chitosan and collagen), which results in the large energy barrier for the rotation of associated groups linked by a double bond (C=N), and finally, this provided the brittle nature to the material.

**Figure 3:**
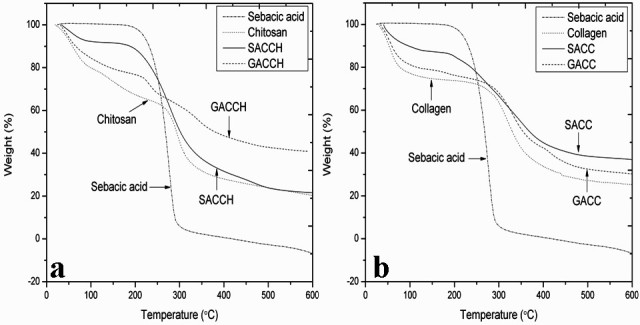
**Thermogravimetric analyses for experimental samples.** (**a**) Thermogravimetric analysis of sebacic acid (SA), chitosan, sebacic acid cross-linked chitosan (SACCH), and glutaraldehyde cross-linked chitosan (GACCH) scaffolds. (**b**) Thermogravimetric analysis of SA, collagen, sebacic acid cross-linked collagen (SACC), and glutaraldehyde cross-linked collagen (GACC) scaffolds.

Thermogravimetric analyses for the experimental samples SA, chitosan, type I collagen, SACCH, SACC, glutaraldehyde cross-linked chitosan (GACCH), and glutaraldehyde cross-linked collagen (GACC) were illustrated in Figure 3a,b, and the corresponding thermal degradation values were displayed in Table 3. From the results, we observed that the incorporation of SA with chitosan and type I collagen tended to shift the thermal region into higher temperature, and such a shift is attributed to an increase in thermal stability.

**Table 3:** **Thermal analyses of SA, chitosan, SACCH, GACCH, collagen, SACC, and GACC under N**_**2**_ **air atmosphere**

Temperature (°C)	Percentage of weight loss (heating rate 20°C/min)						
	SA	Chitosan	SACCH	GACCH	Collagen	SACC	GACC
100	0	21	8	16	23	11	20
200	1	33	12	24	27	15	24
300	95	56	50	38	37	34	33
400	100	73	69	52	67	55	58
500	100	77	77	58	73	62	68
600	100	81	79	60	75	63	70

SA, sebacic acid; SACCH, sebacic acid cross-linked chitosan; GACCH, glutaraldehyde cross-linked chitosan; SACC, sebacic acid cross-linked collagen; GACC, glutaraldehyde cross-linked collagen.

**Figure 4:**
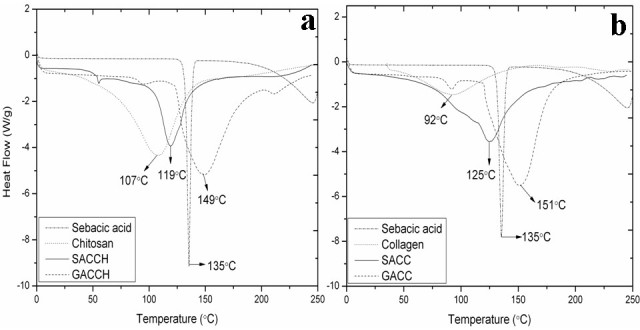
**DSC analyses of SA, chitosan, type I collagen, SACCH, SACC, GACCH, and GACC.** (**a**) DSC analysis of sebacic acid (SA), chitosan, sebacic acid cross-linked chitosan (SACCH), and glutaraldehyde cross-linked chitosan (GACCH) scaffolds. (**b**) DSC analysis of sebacic acid (SA), collagen, sebacic acid cross-linked collagen (SACC), and glutaraldehyde cross-linked collagen (GACC) scaffolds.

Differential scanning calorimetry (DSC) studies were performed to understand the behavior of SACCH and SACC on the application of thermal energy. The thermogram values of SA, chitosan, type I collagen, SACCH, SACC, GACCH, and GACC were shown in Figure 4a,b. DSC studies recorded the melting temperature of SA (135°C) and the degradation temperature differences among chitosan (107°C), type I collagen (92°C), SACCH (119°C), and SACC (125°C), whereas in GACCH and GACC, it was observed at 149°C and 151°C, respectively. The higher transition temperature suggests that SACCH and SACC have high stability at high-temperature environment. Thermal stability also influences on the durability of the scaffolds. A similar kind of observation was reported by Bhumkar and Pokharkar (2006).

**Figure 5:**
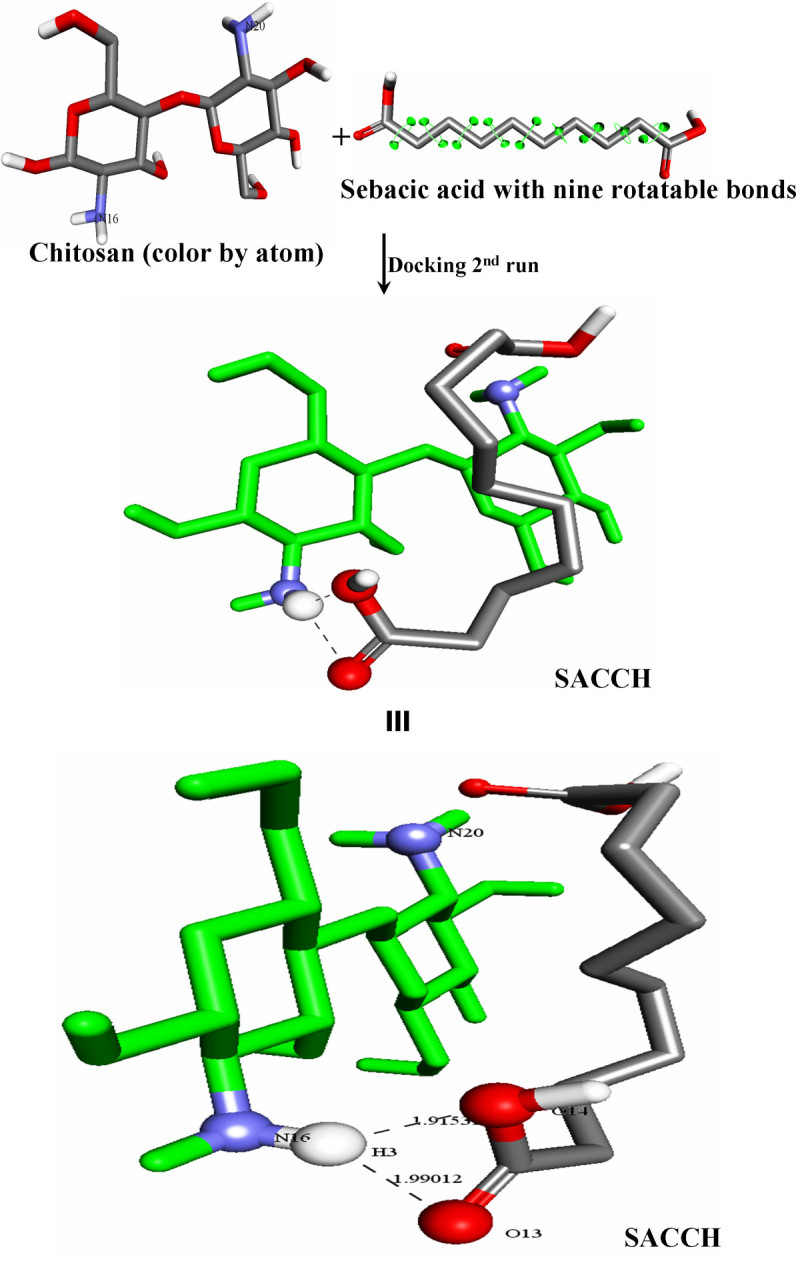
**Multiple hydrogen bonding interactions.** (**a**) Multiple hydrogen bonding interaction between chitosan and sebacic acid. Images of SACCH are displayed in two different orientations. The first orientation demonstrates the interaction, and the second illustrates the details on hydrogen bonding. (**b**) Multiple hydrogen bonding interaction between collagen and sebacic acid. The black dotted line indicates the hydrogen bond. Various colors denote the different atoms to be recognized: white color is for hydrogen atom (H), red color indicates oxygen atom (O), gray is for carbon atom (C), and blue corresponds to nitrogen atom (N).

Results on binding energy calculations based on the bioinformatics tool for the cross-linking of SA with chitosan and type I collagen using AutoDock software (The Scripps Research Institute La Jolla, CA 92037, USA) proved that chitosan and type I collagen cross-links with sebacic acid not only through ionic interaction, but also through multiple intermolecular hydrogen bonding. AutoDock is an automated procedure for predicting the interaction of ligands with biomacromolecular targets. Hundred runs were given for docking SA with chitosan and type I collagen. The best binding energy values and their corresponding rank and run numbers were depicted in Table 4. The binding energy of -4.21 and -4.49 (kcal/mol) was observed when SA interacted with chitosan and type I collagen, respectively. These interactions were made by multiple intermolecular hydrogen bonds between the -COOH group of SA and the -NH_2_ group of chitosan and the free *ε*-NH_2_ group of lysine from type I collagen (Figure 5a,b). In addition to ionic cross-linking, hydrogen bonding interaction also improves the mechanical property of the scaffold. The ionic interaction and hydrogen bonding between the -COOH group of cross-linker and the -NH_2_ group of natural polymers (chitosan/collagen) has already been reported Milosavljevic et al. (2010; Vijayaraghavan et al. 2010). The details of these intermolecular hydrogen bonding sites are given in the following sections.

**Table 4:** **Binding energy values of SACCH and SACC scaffolds**

	Rank	Binding energy (Kcal/mol)	Number of runs
Binding energy calculation between chitosan and sebacic acid based on AutoDock tool software	1	-4.21	2
	2	-4.17	24
	3	-3.92	65
	4	-3.73	29
Binding energy calculation between type I collagen and sebacic acid based on AutoDock tool software	1	-4.49	49
	2	-4.36	20
	3	-4.04	58
	4	-3.94	21
	5	-3.79	51
	6	-3.71	63
	7	-3.63	15
	8	-3.20	85
	9	-3.13	53

### Intermolecular hydrogen bond details between SA and chitosan

H (3) of chitosan is linked to O (14) of SA with a bond distance of 1.91531, and H (3) of chitosan is linked to O (13) of SA with a bond distance of 1.99012.

### Intermolecular hydrogen bond details between SA and type I collagen

Lysine amino acid (LYS) (12) H2 of type I collagen is linked with O (7) of SA with a bond distance of 2.06994, LYS (12) H2 of type I collagen is linked with O (8) of SA with a bond distance of 2.09251, LYS (12) H3 of type I collagen is linked with O (13) of SA with a bond distance of 2.11339, and LYS (12) H3 of type I collagen is linked with O (14) of SA with a bond distance of 1.91386.

**Figure 6:**
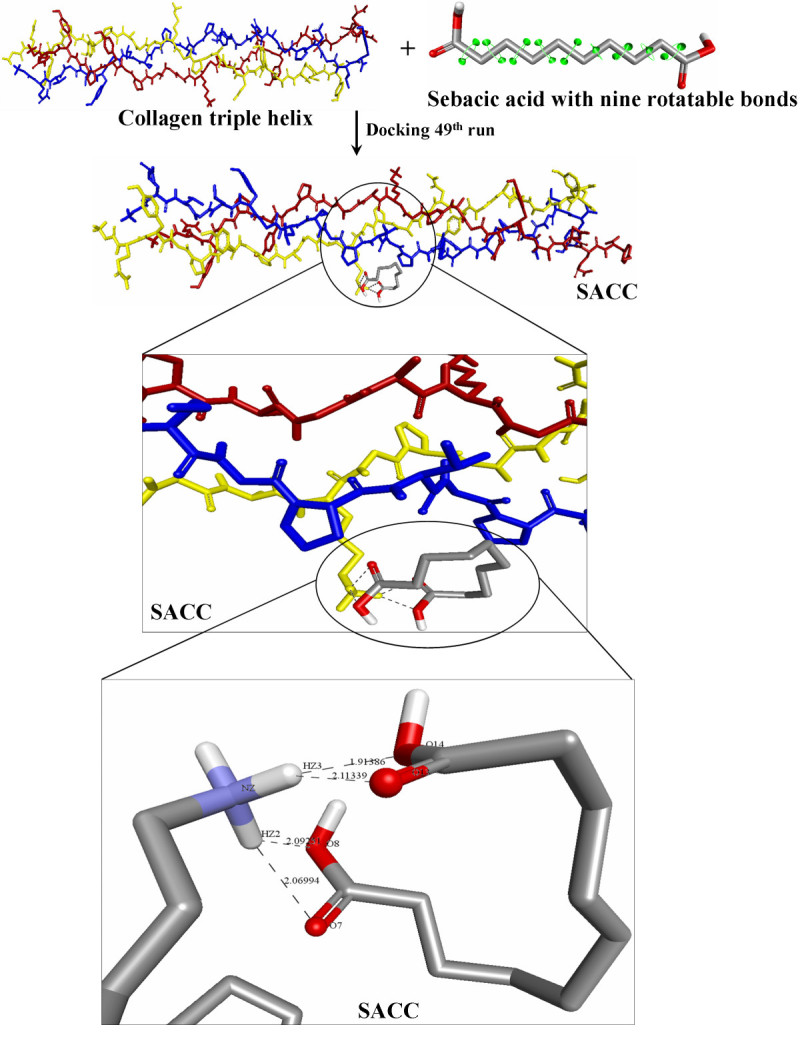
**MTT analysis at 24-, 48-, and 72-h time interval.** The analysis was for control, chitosan, collagen, sebacic acid cross-linked chitosan (SACCH), glutaraldehyde cross-linked chitosan (GACCH), sebacic acid cross-linked collagen (SACC), and glutaraldehyde cross-linked collagen (GACC) scaffolds.

Concerning the biocompatibility of the resulting polymers, cell attachment and viability assays were carried out. 3-[4,5-Dimethylthiazol-2-yl]-2,5-dephenyltetrazolium bromide (MTT) assay was done to check the toxicity profile of the prepared scaffolds (SACCH, SACC, GACCH, and GACC). Only cells that are metabolically normal can turn the tetrazolium salts into purple crystals. Compared with the native chitosan and type I collagen, SACCH and SACC showed no significant differences in absorbance (Figure 6) and inferred that scaffolds being in direct contact with fibroblast did not lead to apoptosis or necrosis. MTT results clearly indicated that NIH 3T3 cells are viable on the surface of the SA cross-linked scaffolds (SACCH and SACC). However, only 10% cells were viable with the samples of GACCH and GACC. Though glutaraldehyde (GA) has been widely used as chemical cross-linking agent Jorge-Herrero et al. (1999) because of stabilizing the collagen efficiently, and the cross-linking is thought to involve the formation of Schiff bases Olde Damink et al. (1995b), the poor biocompatibility of GA-cross-linked biomaterials with some other cell lines, including human fibroblasts, osteoblasts, Chang cells, and endothelial cells, had been reported Gough et al. (2002). The side effects of GA treatment were attributed to the degradation of the GA-derived cross-links and the continuous release of aldehydes contributing to prolong toxic effects Schmidt and Baier (2000).

**Figure 7:**
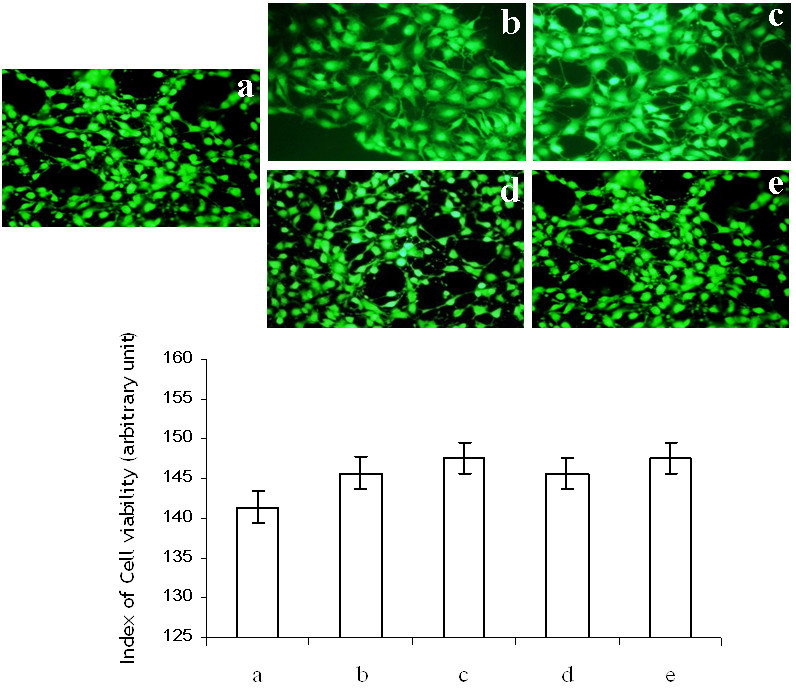
**Cell viability index (arbitrary unit) assessed in SACCH and SACC compared with parent molecules and control.** (**a**) Control, (**b**) chitosan, (**c**) collagen, (**d**) sebacic acid cross-linked chitosan (SACCH), and (**e**) sebacic acid cross-linked collagen (SACC). The assay was carried out using a cell tracker kit. NIH3T3 cells were treated on the surface of native and cross-linked scaffolds for 6 h followed by incubation with cell viable dye cell tracker for 30 min. Fluorescence images of the cells were acquired using DP71 camera adapted to an Olympus IX71 microscope (Olympus Corporation, Shinjuku, Tokyo, Japan). Intensity of green positive cells were counted and plotted. Next, fluorescence intensities of the images were calculated using Adobe Photoshop version 7.0.

In the cell viability assay, we observed intense fluorescence of the cells on the surface of the native and SA cross-linked scaffolds and suggest the viability of the cells as illustrated in Figure 7.

**Figure 8:**
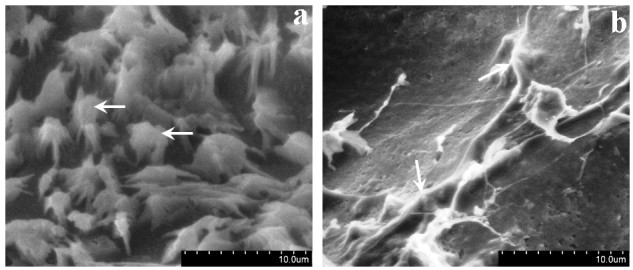
**Attachment of fibroblast cells on the (a) SACCH and (b) SACC scaffolds.** In (**a**), white arrows indicate the adhered cells on the scaffold. In (**b**), porous SACC scaffold was completely covered by fibroblast cells which are indicated by the white arrow.

The SEM images of the cell seeded SACCH and SACC scaffolds displayed in Figure 8a,b demonstrated that after being cultured for a prolonged time (12 days), fibroblasts were detected in the scaffolds (SACCH and SACC) with typical spindle-shaped morphology, suggesting that the cells were infiltrated into the scaffolds.

When comparing the present results with the previous work carried out with malonic acid (three carbons) Mitra et al. (2012), no significant differences in mechanical and thermal stability were observed. However, when comparing the pore size (16 to 18 μm) determination from SEM results of malonic acid cross-linked chitosan/collagen, an increased pore size (27 to 30 μm) was observed in SACCH and SACC scaffolds. This could be due to the long chain length of sebacic acid (ten carbons) compared with malonic acid (three carbons). In addition, the docking studies suggested that there was stronger intermolecular hydrogen bonding interaction between sebacic acid and chitosan/collagen compared with the interaction between malonic acid and chitosan/collagen.

## Conclusions

The present study explicitly demonstrates sebacic acid acting as suitable cross-linker for the preparation of biocompatible scaffolds from natural polymers (chitosan and collagen) with appreciable mechanical properties compared to short-chain dicarboxylic acids. The interactions between SA and chitosan or collagen were identified as non-covalent, i.e., both for ionic and multiple intermolecular hydrogen bonding interactions. These non-covalent interactions offered high mechanical strength to the resultant material. All the instrumental analyses and bioinformatics tool authenticated the non-covalent interactions. The scaffold material prepared upon cross-linking of SA with chitosan or collagen was the green method of preparation. Sebacic acid not only dissolves the above-said natural polymers, but also improves the property of the material through its non-covalent interactions with natural polymers. No toxic compounds were involved in this preparation, and the resultant material can be used as a wound dressing material or as an implant in clinical applications.

## Methods

### Materials

Chitosan from shrimp shells (≥75% deacetylated), sebacic acid, and Picrylsulfonic acid (TNBS) were obtained from Sigma-Aldrich Corporation (St. Louis, MO, USA). MTT and dexamethasone were purchased from HiMedia Laboratories (Mumbai, India). All the other reagents were of analytical grade and used without further purification. Type I collagen from bovine skin was extracted according to the procedure followed by Mitra et al. (2011).

### Preparation of a 3D scaffold

In the powder form of chitosan (1%), sebacic acid at different concentrations was mixed, and 20 ml of water was added to the mixture which was stirred at room temperature until the solution became homogenous. Similarly, collagen (0.5%) and sebacic acid at different concentrations were mixed in the presence of 20 ml of water and stirred at 4°C to obtain a clear homogenous solution. The concentration of SA varied between 0.05% and 0.5% (*w*/*v*). The samples were subjected to centrifugation in order to remove any nonreactive molecules, and a clear solution obtained from centrifugation at 5,000 rpm for 10 min was poured in Tarsons vials (Tarsons Product Pvt. Ltd., Kolkata, India), which have an inner diameter of 4.5 cm and frozen at -4°C for 2 h, -20°C for 12 h, and -80°C for another 12 h according to Peng et al. (2006). The frozen samples were lyophilized for 48 h at a vacuum of 7.5 mTorr (1 Pa) and a condenser temperature of -70°C (PENQU CLASSIC PLUS, Lark, India). The resultant 3D scaffold material was neutralized with repeated washings with 0.05 N of NaOH and ethanol mixture, followed by washings with water and ethanol mixture; finally, it was again lyophilized for 24 h. The scaffolds obtained during this procedure were designated as SACCH and SACC. For comparative analysis, GACCH and GACC were prepared according to the method described previously using 0.2% glutaraldehyde.

### SEM analysis of SACCH and SACC

The physical texture and the morphology of the scaffold of SACCH and SACC were assessed using a scanning electron micrograph. SEM micrograph analysis was made using FEI Quanta (FEI Company, Hillsboro, OR, USA) FEG 200 high-resolution scanning electron microscope under a high voltage at 20 kV.

### FT-IR analysis

Functional group analysis for SA, chitosan, type I collagen, SACCH, and SACC scaffolds were made using Spectrum One FT-IR (PerkinElmer Instruments, Branford, CT, USA). All spectra were recorded with the resolution of 4 cm^-1^ in the range of 400 to 4,000 cm^-1^ with 20 scans.

### Cross-linking degree (2,4,6-trinitrobenzenesulfonic acid assay) determination

Degree of cross-linking was quantified using TNBS assay according to the procedure summarized by Bubnis and Ofner (1992). In brief, native and cross-linked (SACCH and SACC) biopolymer materials were cut into small pieces at 4.5 mm. Cut pieces (6 mg) were immersed in a 2-ml solution (1 ml of 4% disodium hydrogen orthophosphate (*w*/*v*) and 1 ml of 0.5% TNBS (*v*/*v*)) and incubated at 40°C for 2 h. Pure sebacic acid at respective percentages was also treated with a 2-ml solution in separate test tubes. Termination of reaction was by the addition of 3 ml of 6 M HCl (*v*/*v*), and the incubation was continued at 60°C for 90 min. The absorbance of the resulting solution was measured at 345 nm using a UV-visible spectrophotometer (UV-2450, Shimadzu Corporation, Nakagyo-ku, Kyoto, Japan), and the percentage of cross-linking was calculated from the difference in the absorbance divided by the absorbance of the native material and then multiplied by 100.

### Mechanical properties of SACCH, SACC, GACCH, and GACC scaffolds

Mechanical properties, *viz.*, Young's modulus, ultimate tensile strength, stiffness, and percentage of elongation of the dried scaffold materials, were measured using a universal testing machine (model 1405, Instron Corporation, Norwood, MA, USA) at a crosshead speed of 5 mm min^-1^ at 25°C and 65% relative humidity. Length and width of the dumbbell-shaped test samples were maintained at 20 and 5 mm, respectively, according to Shanmugasundaram et al. (2004). All the mechanical tests were performed with dried samples and were examined in triplicates.

### Thermogravimetric analysis

Thermal decomposition analysis of SA, native, and cross-linked scaffolds (chitosan, type I collagen, SACCH, SACC, GACCH, and GACC) was carried out under nitrogen flow (40 and 60 ml min^-1^) with ramp at 20°C min^-1^ using TGA Q 50 (TA Instruments, New Castle, DE, USA) with an isothermal temperature accuracy of ±1°C.

### Differential scanning calorimetry

DSC analysis for SA, native, and cross-linked scaffolds (chitosan, type I collagen, SACCH, SACC, GACCH, and GACC) was analyzed using a differential scanning calorimeter (model DSC Q 200, TA Instruments) with standard mode at nitrogen (50 ml min^-1^) atmosphere with ramp at 10°C min^-1^.

### Docking and binding energy calculations

For the docking study, chemical structures of chitosan and SA were generated using ACD/ChemSketch ACD (2009), and the 3D structure of type I collagen was generated using the gencollagen program. The docking technique is useful to find the binding efficiency with a ligand and a chemical compound. To find out the interaction between chitosan and type I collagen with SA, AutoDock 4.2 was used to calculate Morris et al. (2009) the free energy of binding of SA with chitosan and type I collagen.

### Biocompatibility of SACCH and SACC scaffold an *in vitro* assessment

#### Cell viability study (MTT assay)

NIH 3T3 fibroblast cells were grown in Dulbecco's modified Eagle's medium supplemented with 10% fetal bovine serum (*v*/*v*) and 1% antibiotics and were incubated at 37°C in 5% CO_2_ humidified atmosphere. Polystyrene 96-well culture plates (Tarsons) were coated with native chitosan, type I collagen, SA cross-linked chitosan (SACCH), GA cross-linked chitosan, SA cross-linked type I collagen (SACC), and GA cross-linked collagen samples. The plates were dried using a laminar air flow hood, followed by 30 min of UV sterilization. The cells were seeded at the density of 0.5 × 10^6^ per well and incubated at 37°C in humidified atmosphere containing 5% CO_2_. At scheduled time points of 24, 48, and 72 h, the supernatant of each well was replaced with MTT diluted in serum-free medium, and the plates incubated at 37°C for 4 h. After removing the MTT solution, acid isopropanol (0.04 N HCl in isopropanol) was added to each well, was then pipetted up and down to dissolve all the dark-blue crystals, and was left at room temperature for a few minutes to ensure the dissolution of all crystals. Finally, absorbance was measured at 570 nm using a UV spectrophotometer Mossmann (1983). Each experiment was performed at least three times. The sets of three wells for the MTT assay were used for each experimental variable.

#### Cell tracker assay to detect live cells

Cell viability was measured using 5-chloromethylfluorescein diacetate probe (CMFDA) (Invitrogen Life Technologies, Carlsbad, CA, USA). NIH 3T3 cells were subjected to respective treatment conditions. Cells were probed with 5 μM CMFDA and incubated for 2 h. Cells were then washed with sterile PBS, and images were taken using DP71 camera adapted to an Olympus IX71 (Olympus Corporation) microscope Majumder et al. (2011). The assay was carried out using a cell tracker kit. NIH3T3 Cells were treated on the surface of native and cross-linked scaffolds for 6 h, followed by the incubation with cell viable dye cell tracker for 30 min. Fluorescence images of the cells were acquired using a DP71 camera adapted to an Olympus IX71 microscope. The intensity of green positive cells was counted and plotted. Next, fluorescence intensities of the images were calculated using Adobe Photoshop version 7.0. No cell tracker assay was carried out for GACCH and GACC based on the observations made with the MTT assay.

#### Cell morphology of NIH 3T3 cells in SACCH and SACC scaffolds

SACCH and SACC scaffolds (2 × 2 × 1 cm) were placed individually in six-well culture plates (Tarsons, India) and sterilized ETO. Culture media were added to the scaffolds overnight. NIH 3T3 fibroblast cells were seeded onto the scaffolds at a density of 5 × 10^4^ cells and incubated in an atmosphere of 5% CO_2_ at 37°C. The medium was changed every 24 h. The morphology of cells was examined after 12 days according to the following procedure:

1. The cells-and-scaffold constructs were fixed in 2.5% glutaraldehyde and dehydrated through a graded ethanol series.
2. The dried cells-and-scaffold were coated with gold (E-1010 Ion sputter, Hitachi High-Tech, Minato-ku, Tokyo, Japan) and examined under SEM (S-3400 N Hitachi High-Tech).

Experiments for GACCH and GACC were not conducted.

#### Statistical analysis

When necessary, the experimental results were expressed as the mean ± SD values of triplicates.

## Authors’ original submitted files for images

Below are the links to the authors’ original submitted files for images. Authors’ original file for figure 1 Authors’ original file for figure 2 Authors’ original file for figure 3 Authors’ original file for figure 4 Authors’ original file for figure 5 Authors’ original file for figure 6 Authors’ original file for figure 7 Authors’ original file for figure 8 Authors’ original file for figure 9
